# Tolerance of engineered *Rhodosporidium toruloides* to sorghum hydrolysates during batch and fed-batch lipid production

**DOI:** 10.1186/s13068-023-02429-6

**Published:** 2023-11-29

**Authors:** William Woodruff, Narendra Naik Deshavath, Vionna Susanto, Christopher V. Rao, Vijay Singh

**Affiliations:** 1 Department of Chemical and Biomolecular Engineering, University of Illinois at Urbana-Champaign, Urbana, USA; 2 Department of Agricultural and Biological Engineering, University of Illinois at Urbana-Champaign, Urbana, USA; 3 Center for Advanced Bioenergy and Bioproducts Innovation, University of Illinois at Urbana-Champaign, Urbana, USA

**Keywords:** Bioenergy sorghum, Citrate buffer, Fermentation, Lipids, Oleaginous yeast, *Rhodosporidium toruloides*

## Abstract

**Background:**

Oleaginous yeasts are a promising candidate for the sustainable conversion of lignocellulosic feedstocks into fuels and chemicals, but their growth on these substrates can be inhibited as a result of upstream pretreatment and enzymatic hydrolysis conditions. Previous studies indicate a high citrate buffer concentration during hydrolysis inhibits downstream cell growth and ethanol fermentation in *Saccharomyces cerevisiae*. In this study, an engineered *Rhodosporidium toruloides* strain with enhanced lipid accumulation was grown on sorghum hydrolysate with high and low citrate buffer concentrations.

**Results:**

Both hydrolysis conditions resulted in similar sugar recovery rates and concentrations. No significant differences in cell growth, sugar utilization rates, or lipid production rates were observed between the two citrate buffer conditions during batch fermentation of *R. toruloides*. Under fed-batch growth on low-citrate hydrolysate a lipid titer of 16.7 g/L was obtained.

**Conclusions:**

Citrate buffer was not found to inhibit growth or lipid production in this engineered *R. toruloides* strain, nor did reducing the citrate buffer concentration negatively affect sugar yields in the hydrolysate. As this process is scaled-up, $131 per ton of hydrothermally pretreated biomass can be saved by use of the lower citrate buffer concentration during enzymatic hydrolysis.

**Graphical Abstract:**

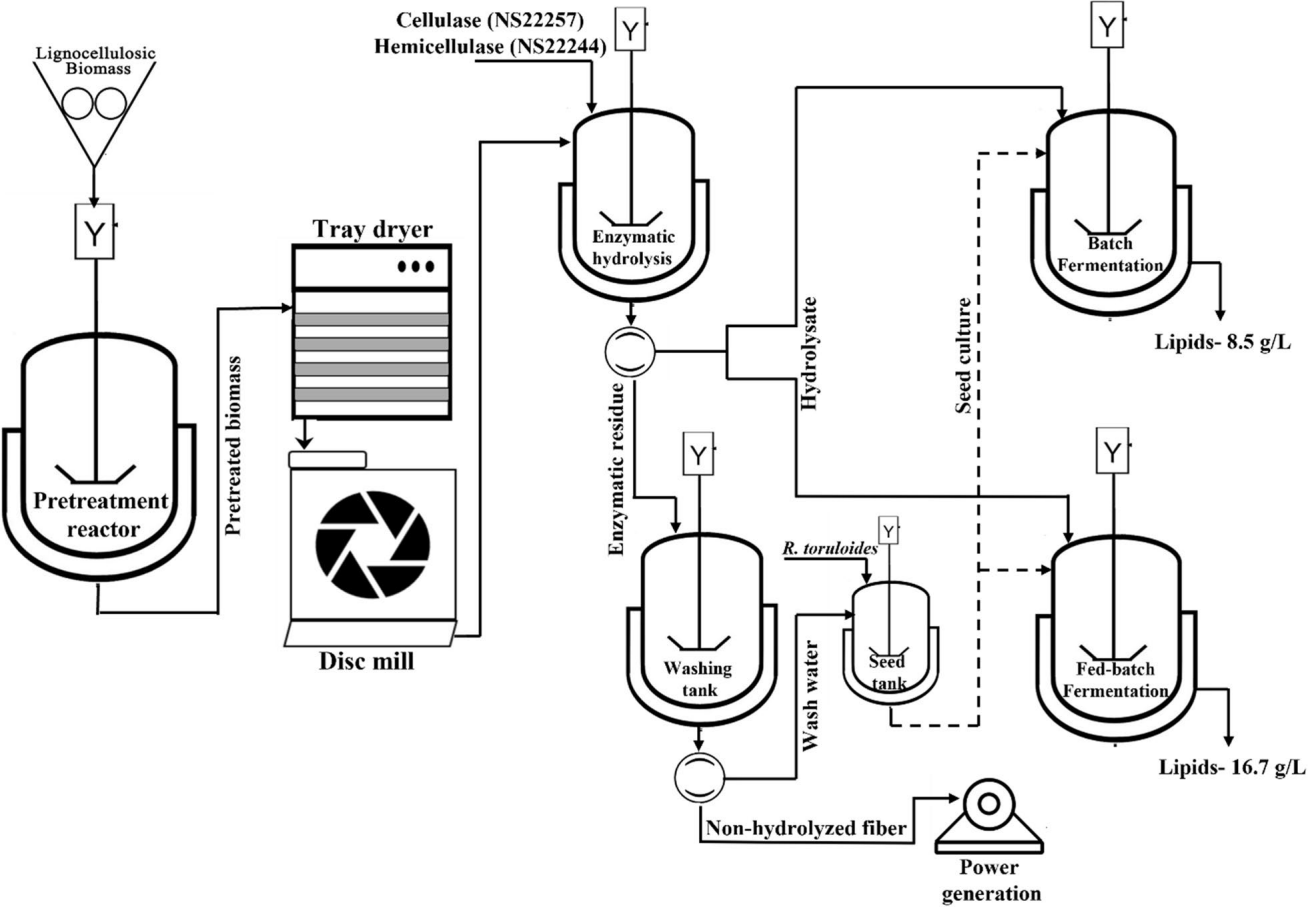

**Supplementary Information:**

The online version contains supplementary material available at 10.1186/s13068-023-02429-6.

## Background

The conversion of lignocellulosic substrates into fuels and chemicals is a key technology needed to develop a sustainable bioeconomy. The rising environmental and energy costs of petroleum extraction and refining in particular incentivize the development of alternative hydrocarbon sources. Plant and microbial oils are the closest biological analogues to fossil fuels and have long been explored as a potential replacement for these finite resources [1–3]. Oleaginous yeasts such as *Rhodosporidium toruloides* (alt. *Rhodotorula toruloides*) are promising candidates for large-scale microbial oil production, as they can be grown on a wide variety of lignocellulosic substrates and accumulate over 70% of their dry cell weight in lipids [4–6].

The major components of lignocellulosic biomass are cellulose, hemicellulose, and lignin polymers [7–9]. Pretreatment and subsequent enzymatic hydrolysis of lignocellulosic biomass release hexose and pentose sugars from cellulose and hemicellulose, aromatic subunits from lignin, and organic acids and furans that may inhibit microbial fermentation [10, 11]. Control of pH with buffers such as sodium citrate is necessary to maintain enzymatic activity during the hydrolysis of pretreated biomass. However, using excess buffer is an added material cost and may inhibit subsequent microbial growth during fermentation [11].

Sorghum is a leading candidate bioenergy crop due to its high yield, drought tolerance, and the availability of gene editing tools [12]. Hybrid varieties with superior qualities for ethanol and biodiesel production have been developed and tested under various pretreatment and extraction conditions [13–15]. Dilute acid pretreatment, the most widely employed method, requires additional downstream neutralization and detoxification steps and corrosion-resistant equipment [12, 16]. Hydrothermal pretreatment is an alternative process which is less harsh and generates negligible concentrations of sugar decomposition products while permitting downstream enzymatic hydrolysis at a lower buffer concentration [10].

Previous studies indicate that cell growth and ethanol production in the model yeast *Saccharomyces cerevisiae* on hydrothermally pretreated sorghum hydrolysate are inhibited at higher citrate buffer concentrations [11, 17]; however, non-model oleaginous yeasts such as *R. toruloides* are more tolerant of fermentation inhibitors and are natively capable of degrading all major components of lignocellulose [5, 6, 18]. The development of advanced gene editing tools for *R. toruloides* in recent years has enabled the creation of strains engineered for superior lipid and oleochemical production [19–21].

In this study, a previously engineered *R. toruloides* strain with enhanced intracellular triacylglycerol (TAG) accumulation was grown in bioreactors on sorghum hydrolysate prepared via hydrothermal pretreatment and enzymatic hydrolysis at high and low citrate buffer concentrations [11, 17, 21, 22]. Wash water prepared from enzymatic hydrolysis residue was tested as a seed culture medium and lipid production was evaluated in both batch and fed-batch modes in lab-scale bioreactors.

## Results

### Hydrothermal pretreatment

**Fig. 1:**
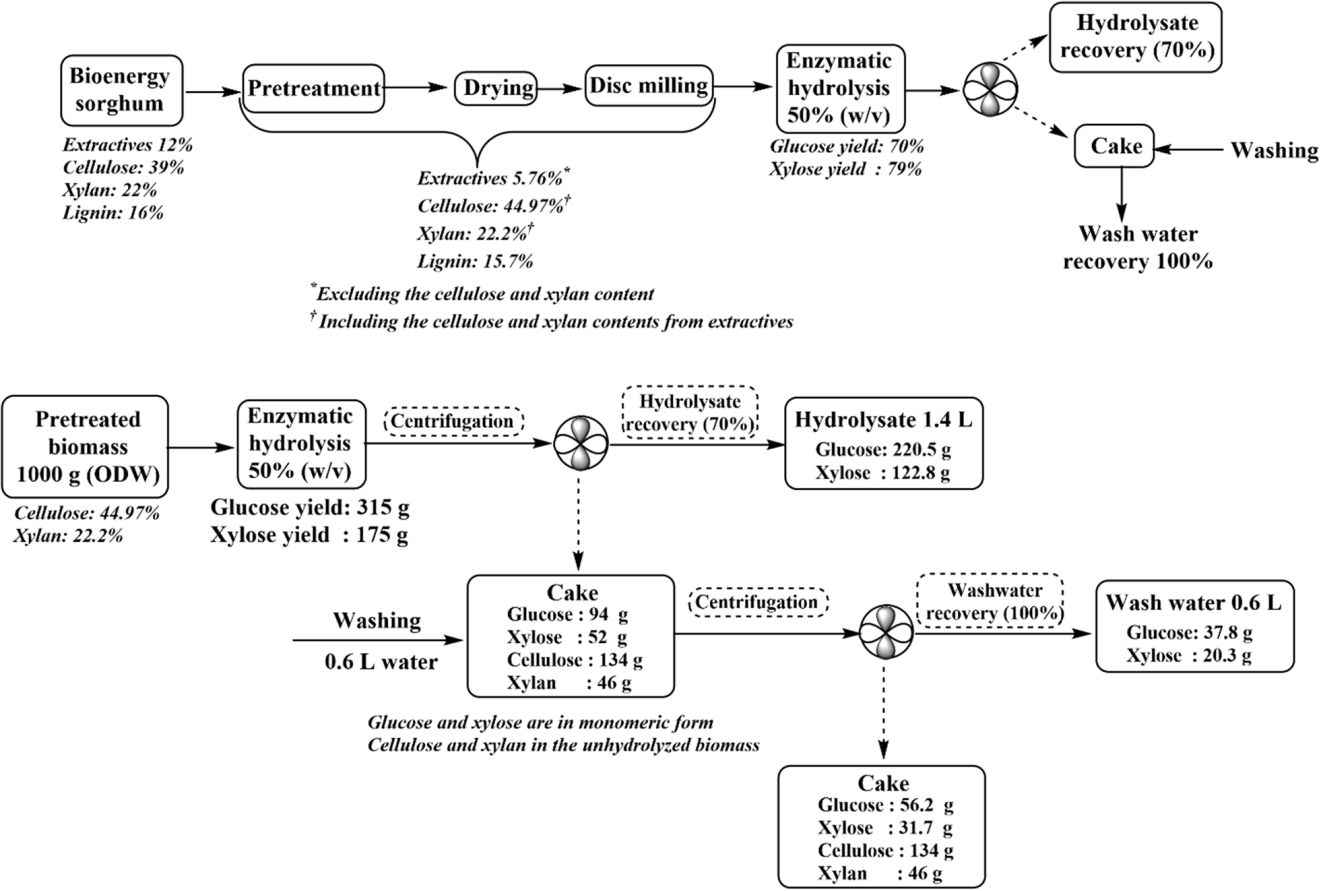
Schematic of sorghum biomass processing steps including hydrothermal pretreatment, enzymatic hydrolysis, and the overall mass balance per kg of pretreated biomass

Previous studies indicate that hydrothermal pretreatment followed by disc milling reduces the recalcitrance of bioenergy sorghum biomass without greatly impacting the overall composition [11]. In accordance with these results, the pretreated biomass contained 44.97% glucan and 22.2% xylan, compared to 39% glucan and 22% xylan in the untreated biomass (Fig. 1). The majority of the xylan and a large fraction of cellulose were solubilized during the pretreatment process, while most of the lignin remained in the solid fraction.

### Enzymatic hydrolysis

Reduced citrate buffer concentration was previously shown to not substantially impact sugar concentrations obtained through enzymatic hydrolysis of hydrothermally pretreated sorghum biomass, as this pretreatment does not generate as many fermentation inhibitors as the standard dilute acid pretreatment process [11]. In this study, fed-batch enzymatic hydrolysis was performed using 0.5 mM and 50 mM citrate buffer at 50% solid loading. Similar final sugar and inhibitor concentrations in both hydrolysate and wash water were observed under these two conditions (Table 1). The average glucose and xylose yields in the hydrolysate were 137.15 ± 0.08 g/L and 77.95 ± 0.75 g/L, respectively.

**Table 1:** Composition of enzymatic hydrolysates at 50% (w/v) solid loading

Citrate buffer (mM)	Cellobiose (g/L)	Glucose (g/L)	Xylose (g/L)	Furfural (g/L)	Acetic acid (g/L)	Formic acid (g/L)
50	6.55 ± 0.63	137.22 ± 0.17	78.48 ± 0.51	0.07 ± 0.02	6.69 ± 0.34	3.55 ± 0.17
0.5	7.72 ± 0.07	137.1 ± 0.15	77.42 ± 0.14	0.07 ± 0.02	6.51 ± 0.12	3.61 ± 0.05
50 (ww)^a^	4.01 ± 0.42	65.65 ± 3.47	36.58 ± 3.82	0.02 ± 0.01	3.06 ± 0.18	1.32 ± 0.08
0.5 (ww)^a^	3.41 ± 0.74	67.44 ± 2.74	37.45 ± 3.31	0.029 ± 0.01	3.13 ± 0.09	1.36 ± 0.08

^a^*WW* wash water

Average ± standard deviation

Seventy percent of the initial volume of enzymatic hydrolysis medium was recovered as liquid hydrolysate following centrifugation (0.42 L, containing 57.1 g of glucose and 32.6 g of xylose). From the remaining 30% of the liquid remaining within the enzymatic residue (0.18 L, containing 24.5 g of glucose and 14.0 g of xylose), about 40% of the sugar (11.6 g of glucose and 6.21 g of xylose) was recovered following the additional wash step. This wash water (0.18 L containing 64.35 ± 1.62 g/L glucose and 34.5 ± 0.84 g/L of xylose) contained enough sugar to be used as a seed culture medium for *R. toruloides*. These same proportions are indicated on a per kg basis of pretreated biomass in the mass balance shown in Fig. 1, which yields 1.4 L of hydrolysate containing 220.5 g of glucose and 112.8 g of xylose and 0.6 L of wash water containing 37.8 g of glucose and 20.3 g of xylose. Additionally, carbon, hydrogen, and nitrogen (CHN) and inductively coupled plasma (ICP) analyses were performed on triplicate samples of sorghum hydrolysate prepared with 50 mM citrate buffer to determine elemental and micronutrient compositions. A C/N mass ratio of 63 was calculated from these results (Additional file 1: Fig. S1).

### Cell culture and batch fermentation

*R. toruloides* strain RT-ADS was previously obtained from the base strain IFO0880 by overexpressing three genes: acetyl-CoA carboxylase (ACC1), diacylglycerol acyltransferase (DGA1), and stearoyl-CoA desaturase (SCD) (Additional file 1: Fig. S2, Table S1) [22]. These modifications increased the overall production of triacylglycerol (TAG) within the cell, and this strain has been shown to produce up to 27.4 g/L of lipid during batch growth on low-nitrogen glucose media [22]. To prepare seed cultures, RT-ADS cells were streaked on selective plates and individual colonies inoculated in YPD media for overnight growth. The next day, flasks containing 25 mL of seed media at a total sugar concentration of 40 g/L were prepared in duplicate for each citrate buffer concentration and RT-ADS cells were inoculated at an initial OD_600_ of 0.5. This second seed culture step was performed to adapt the cells to the biomass substrate at a lower concentration and to obtain sufficient cell mass for inoculation of the bioreactors. After 48 h of growth, cells from each flask were washed and transferred to a bioreactor containing 150 mL of sorghum hydrolysate media diluted to a total sugar concentration of 80 g/L, and inoculated at an initial OD_600_ of 1.0.

**Fig. 2:**
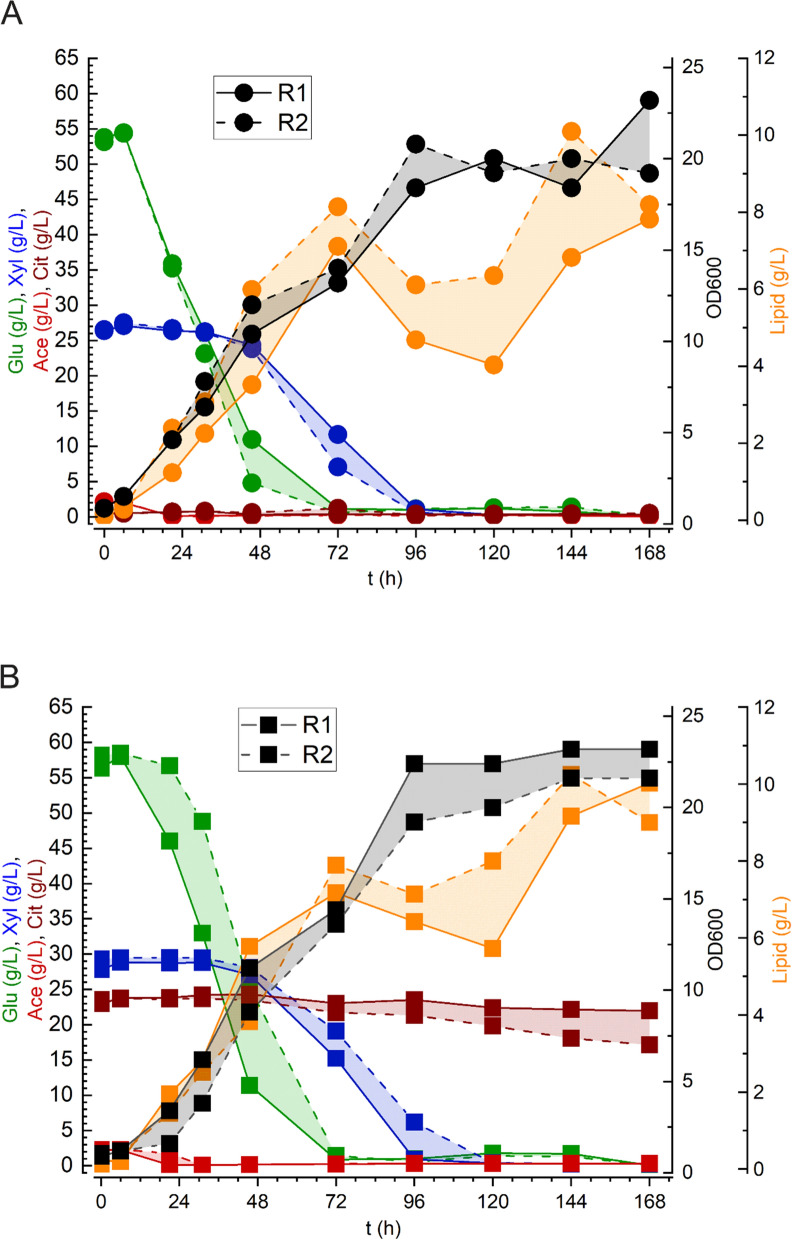
Batch reactor growth curves for RT-ADS cells grown on diluted sorghum hydrolysate prepared using 0.5 mM (**A**) or 50 mM (**B**) citrate buffer. The cultures were maintained at 30 °C, 30% DO and pH 5.6. The dotted and solid lines represent the results of two independent experiments

The resulting growth profiles of RT-ADS on the two hydrolysate conditions were quite similar, with a slightly higher final biomass in the 50 mM condition. Across both conditions, glucose was exhausted by 72 h and xylose was exhausted by 120 h. Little or no consumption of xylose was observed in the first 48 h, while the majority of glucose was consumed; however, the small amount of acetate in the media was consumed concurrently with glucose early on. Biomass accumulation was faster during growth on glucose across all conditions as compared to xylose, with a specific growth rate of 0.052 ± 0.003 h^−1^ on glucose and 0.013 ± 0.002 h^−1^ on xylose, and lipid titers decreased transiently during the transition between glucose and xylose consumption before later recovering. In the high-citrate condition, the citrate concentration in the media began to slowly decrease after 48 h of growth, indicating that the cells may have begun to consume it as an additional carbon source. An average lipid titer of 9.7 g/L was obtained from the cells grown on the high-citrate hydrolysate and a slightly lower titer of 8.5 g/L was obtained from the low-citrate condition (Fig. 2). This difference was not statistically significant (*p* = 0.46 as determined using a two-sided t-test). Rates of glucose utilization were much higher than for xylose across all conditions, and lipid production rates and yields were similar to those previously reported for this strain (Table 2) [21, 22]. Xylose is known to be converted into arabitol in *R. toruloides*, bypassing the canonical assimilation pathway and leading to less efficient substrate utilization [23, 24].

**Table 2:** Sugar utilization and lipid production rates for *R. toruloides* cells across all growth conditions

Citrate buffer (mM)	Growth mode	Glucose utilization rate (g/L/h)	Xylose utilization rate (g/L/h)	Lipid production rate (g/L/h)	Lipid yield (g/g sugar)
50 (R1)	Batch	0.42	0.11	0.06	0.11
50 (R2)	Batch	0.38	0.13	0.06	0.10
0.5 (R1)	Batch	0.40	0.11	0.05	0.10
0.5 (R2)	Batch	0.41	0.11	0.06	0.10
0.5 (R1)	Fed-Batch	0.34	0.19	0.08	0.20
0.5 (R2)	Fed-Batch	0.38	0.22	0.07	0.16

Sugar utilization and lipid production rates were averaged for each experiment across all timepoints not including the feeding period

### Fed-batch fermentation

**Fig. 3:**
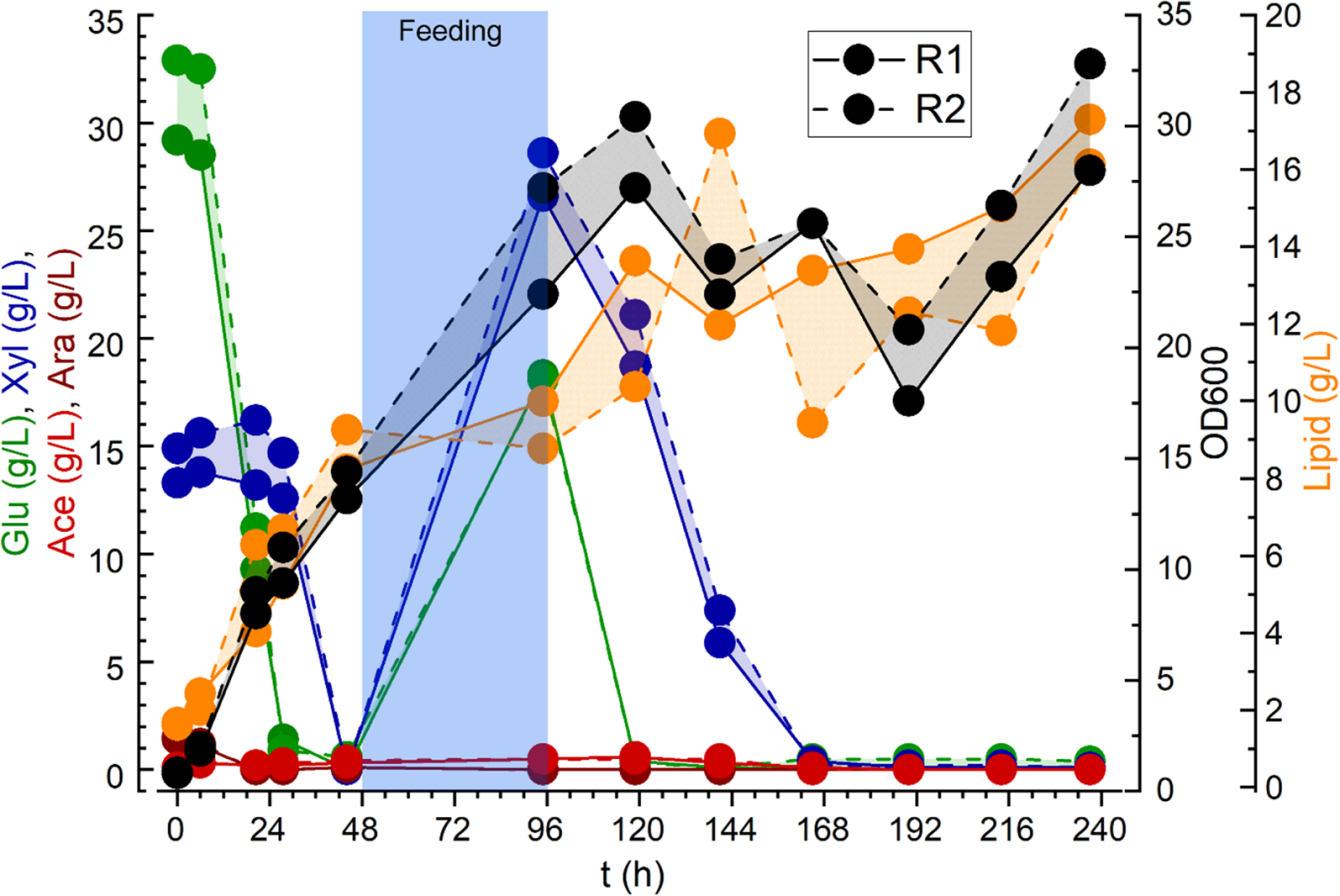
Growth curve for RT-ADS cells grown in fed-batch mode on diluted sorghum hydrolysate prepared using 0.5 mM citrate buffer. The cultures were maintained at 30 °C, 30% DO and pH 5.6. The dotted and solid lines represent the results of two independent experiments

An additional fed-batch fermentation was performed using low-citrate buffer hydrolysate media. The initial sugar concentration in the media was 40 g/L and feed media at a concentration of 120 g/L was added starting at 48 h based on the DO level within the reactors. When DO rose above the set point of 30%, feed media was added at 5 mL/hr. All of the feed media were added by 96 h. A final average OD_600_ of 30.4 and lipid titer of 16.7 g/L was achieved by 237 h (Fig. 3). As in the batch fermentation, glucose utilization rates were higher than for xylose and a transient decrease in biomass and lipid accumulation were observed during the transition to xylose consumption; however, the overall lipid production rate and yield were higher in the fed-batch condition (Table 2).

## Discussion

While previous studies have shown up to a 20% decrease in ethanol yield in *S. cerevisiae* grown in flasks on hydrolysate prepared with 50 mM citrate buffer as compared to 0.5 mM, no significant inhibitive effect of citrate buffer on cell density, growth rates, or lipid titer in *R. toruloides* strain RT-ADS grown in bioreactors was observed in this study (Fig. 2; Table 2) [17]. This is in agreement with observations of strain Y-6987 grown in microtiter plates [11]. Sugar concentrations resulting from enzymatic hydrolysis at low and high-citrate buffer concentrations were also in accordance with previous results, while yields were substantially improved by the additional wash step [11]. As 0.29 g of sodium citrate is required per kg of biomass to prepare buffer at a 0.5 mM concentration, as opposed to 29.4 g for a 50 mM concentration, a bulk cost savings of $131 per ton of biomass was calculated for use of the lower concentration [25]. Both hydrolysate and hydrolysis residue wash water supported robust growth of *R. toruloides* in seed culture or bioreactors. Citrate may have been utilized in low quantities as a carbon source to enhance *R. toruloides* growth in the high-citrate condition, though the overall difference in biomass accumulation was minimal.

Lipid titers were similar between the two citrate buffer conditions, but overall were lower than those previously reported for this engineered strain [21, 22]. Continued accumulation of lipids after the exhaustion of xylose in the media may have resulted from consumption of additional minor hydrolysate components such as cellobiose or formate, or from repurposing of intracellular components through autophagic pathways as a response to starvation conditions, as these pathways are closely linked to lipid metabolism [26]. As sorghum hydrolysate contains residual nitrogen and fermentation inhibitors, it is not optimized for lipid production in *R. toruloides*, and two-phase growth with discrete biomass and lipid accumulation stages was not achieved in the fed-batch fermentation due to the high nutrient levels in the hydrolysate media. While the C/N ratio determined for sorghum hydrolysate in this study was 63, much higher values are typically used for lipid production in *R. toruloides*, with strain-specific optimization often necessary [21, 21, 27, 28]. Additionally, xylose has been shown to be inefficiently metabolized in *R. toruloides* with substantial flux to arabitol rather than biomass or lipids, and this is supported by the lower consumption rate and reduced biomass and lipid accumulation observed once glucose in the media was exhausted [23, 24]. Nevertheless, complete utilization of glucose and xylose was observed and the viability of lipid production using engineered *R. toruloides* on sorghum hydrolysate media prepared with low citrate buffer concentrations has been demonstrated.

## Conclusions

Though it is not necessary to reduce citrate buffer concentration during enzymatic hydrolysis for the purpose of facilitating growth and lipid production in *R. toruloides*, the minimal difference between the two conditions indicates that moving towards a combination of hydrothermal biomass pretreatment followed by low-citrate hydrolysis has the potential to reduce materials cost in the upstream bioprocessing without adversely affecting downstream product yields. Additional work is required to develop new strains of *R. toruloides* and other oleaginous yeasts with enhanced utilization of xylose and production of high-value lipid-derived compounds from biomass.

## Methods

### Feedstock processing and pretreatment

Bioenergy sorghum was collected from the Energy Farm at the University of Illinois at Urbana-Champaign. Biomass was dried to a < 10% (*w/w*) moisture content at room temperature, chopped into 3–4 cm pieces, and ground in a hammer mill to reduce the particle size using a 3 mm sieve. The dry sorghum biomass was rehydrated to 50% (*w/w*) moisture content and run through a pilot-scale continuous hydrothermal pretreatment reactor system (SüPR∙2G Reactor, AdvanceBio LLC., Milford, OH, USA) at 190 °C for 10 min. The pretreated sorghum biomass was dried at 46 ± 3 °C in a tray dryer to a moisture content of < 20% and run through a disc mill to further reduce the particle size.

### Enzymatic hydrolysis

The pretreated sorghum biomass was hydrolyzed in duplicate as previously described [11]. Briefly, fed-batch enzymatic hydrolysis was performed in 2 L screw cap conical flasks containing a final mass of 300 g hydrothermally pretreated solids in 0.6 L of liquid medium at a concentration of 0.5 mM or 50 mM citrate buffer. Cellulase NS22257 and hemicellulase NS22244 (Novozymes North America, Inc., Franklinton, NC, USA) were added at concentrations of 60 mg of cellulase protein/g cellulose and 20 mg of hemicellulase protein/g hemicellulose. Protein concentrations were determined via bicinchoninic acid (BCA) assay (Pierce™ BCA Protein Assay Kit, Thermo Fisher Scientific™, Waltham, MA, USA). Hydrolysis was conducted at 50 °C with 160 rpm mixing for 72 h, with periodic sampling for sugar quantification. Hydrolysate was separated from the solid residue by centrifugation at 8000 rpm for 10 min. The remaining solids were washed with 0.18 L distilled water, agitated at 150 rpm for 10 min, and centrifuged again to recover residual sugars. This wash water was set aside for seed culture preparation.

### Yeast cell culture and bioreactor conditions

YPD media was prepared containing 10 g/L yeast extract, 20 g/L peptone, and 20 g/L glucose. Seed media was prepared using hydrolysis residue wash water diluted to a total sugar concentration (including glucose, xylose, and arabinose) of 40 g/L, titrated to a pH of 5.6 using 1 M KOH, buffered with 25 mM K_2_HPO_4_ and 25 mM NaH_2_PO_4_, and with added YNB salts (MilliporeSigma, Burlington, MA; Y1251) and 4 g/L yeast extract. Batch reactor media was prepared using sorghum hydrolysate prepared using either 0.5 mM or 50 mM citrate buffer diluted to a total sugar concentration of 80 g/L with added YNB salts and 4 g/L yeast extract. For fed-batch growth, batch media was prepared with sorghum hydrolysate prepared using 0.5 mM citrate buffer diluted to a total sugar concentration of 40 g/L with added YNB salts and 4 g/L yeast extract, and feed media was prepared using the same hydrolysate diluted to a total sugar concentration of 120 g/L.

*R. toruloides* strain RT-ADS was previously generated from IFO0880 [21, 22]. Cells were streaked on selective media (YPD agar plates containing 200 µg/mL nourseothricin) from a frozen glycerol stock, grown for 2 days in a 30 °C incubator, and individual colonies were inoculated in 4 mL YPD media (containing 100 µg/mL nourseothricin) in 12 mL conical tubes and grown overnight at 250 RPM and 30 °C. Cells were transferred to 25 mL of seed culture media in 125-mL flasks, diluted to an initial OD_600_ of 0.5, and grown for 48 h under the same conditions.

Batch bioreactor cultures (DASbox Mini Bioreactor System; Eppendorf North America, Enfield, CT) were prepared in duplicate for each citrate buffer concentration with 150 mL of media in each. The airflow rate was 1 vvm, DO was controlled at 30% with agitation, temperature was controlled at 30 °C, and pH was controlled at 5.6 with 4 M HCl and 4 M KOH. Cells were inoculated from seed cultures at an initial OD_600_ of 1.0, grown for 168 h in total, and 1-mL samples were collected at regular intervals for cell density, sugar, and lipid analysis.

For fed-batch growth, bioreactors were prepared in duplicate and inoculated from seed cultures in the same way, with an initial media volume of 75 mL and 125 mL feed media per reactor. Feeding was controlled by a program that added media at a rate of 5 mL/h when DO rose above the set point of 30%. This program was initiated 48 h after inoculation, and all of the feed media was added by 96 h. The flasks were grown for 237 h in total, and 1 mL samples were collected at regular intervals for cell density, sugar, and lipid analysis.

### Sugar consumption and lipid analysis

OD_600_ for each time point was measured using a cell density meter (Ultrospec 10, Biochrom US, Holliston, MA, USA). Sugar and organic acid concentrations were measured using an HPLC (Waters e2695 Separation Module, Waters Corporation, Milford, MA, USA) with a 2414 Refractive Index Detector (RID) and Aminex HPX-87H column (300 × 78 mm, 9 µm particle size; Bio-Rad Laboratories, Hercules, CA, USA) at 65 °C and 5 mM H_2_SO_4_ running buffer at a 0.6 mL/min flow rate. The RID detector was maintained at 30 °C. Lipid titers were determined using a vanillin-phosphoric acid colorimetric assay as previously described (23). Briefly, washed cell pellets were mixed with 0.4 mL 18 M H_2_SO_4_, reacted in glass vials at 100 °C for 10 min in a dry heating bath, and cooled for 10 min at room temperature. 1 mL of freshly prepared vanillin-phosphoric acid was added, and the vials shaken at 37 °C and 150 rpm for 15 min in darkness. After cooling for 10 min, absorbance at 530 nm was measured on a microplate reader to quantify lipids (Safire^2^; Tecan Group Ltd., Männedorf, Switzerland).

### Supplementary Information

**Additional file 1: Figure S1.** Elemental abundances obtained through CHN (Exeter Analytical CE 440) and ICP microanalysis (PerkinElmer NexION 350D) of sorghum hydrolysate prepared using 50 mM citrate buffer. The calculated C/N mass ratio was 63. **Figure S2.** Pathway diagram of glucose and xylose utilization and conversion to lipids in *R. toruloides*. Enzymes overexpressed in the RT-ADS strain are labeled in red. **Table S1.** Strain information

## Data Availability

The datasets generated in this study are available from the corresponding author on reasonable request.
