# Supplementary material for: Tolerance of engineered Rhodosporidium toruloides to sorghum hydrolysates during batch and fed-batch lipid production
Source: Biotechnol Biofuels Bioprod. 2023 Nov 29;16:187. doi: 10.1186/s13068-023-02429-6 (PMC10688463; doi:10.1186/s13068-023-02429-6)
Supplement: Supplementary file 1 — Additional file 1: Figure S1. Elemental abundances obtained through CHN (Exeter Analytical CE 440) and ICP microanalysis (PerkinElmer NexION 350D) of sorghum hydrolysate prepared using 50 mM citrate buffer. The calculated C/N mass ratio was 63. Figure S2. Pathway diagram of glucose and xylose utilization and conversion to lipids in R. toruloides. Enzymes overexpressed in the RT-ADS strain are labeled in red. Table S1. Strain information [file 13068_2023_2429_MOESM1_ESM.docx]

**Effect of Citrate Buffer Concentration on Sorghum Biomass Conversion and Lipid Production of Engineered *Rhodosporidium toruloides* in Bioreactors – Supplement**

William Woodruff, Narendra Naik Deshavath, Vionna Susanto, Christopher V. Rao, Vijay Singh

Freeze-dried and weighed samples of sorghum hydrolysate prepared in triplicate using 50 mM citrate buffer were analyzed to determine elemental abundances of carbon, hydrogen, and nitrogen through thermal conductivity analysis after combustion in pure oxygen in an Exeter Analytical CE 440. Additional samples were prepared in triplicate for inductively coupled plasma (ICP) analysis, where the abundances of several additional pre-selected elements were determined by m/z ratios in a PerkinElmer NexION 350D.


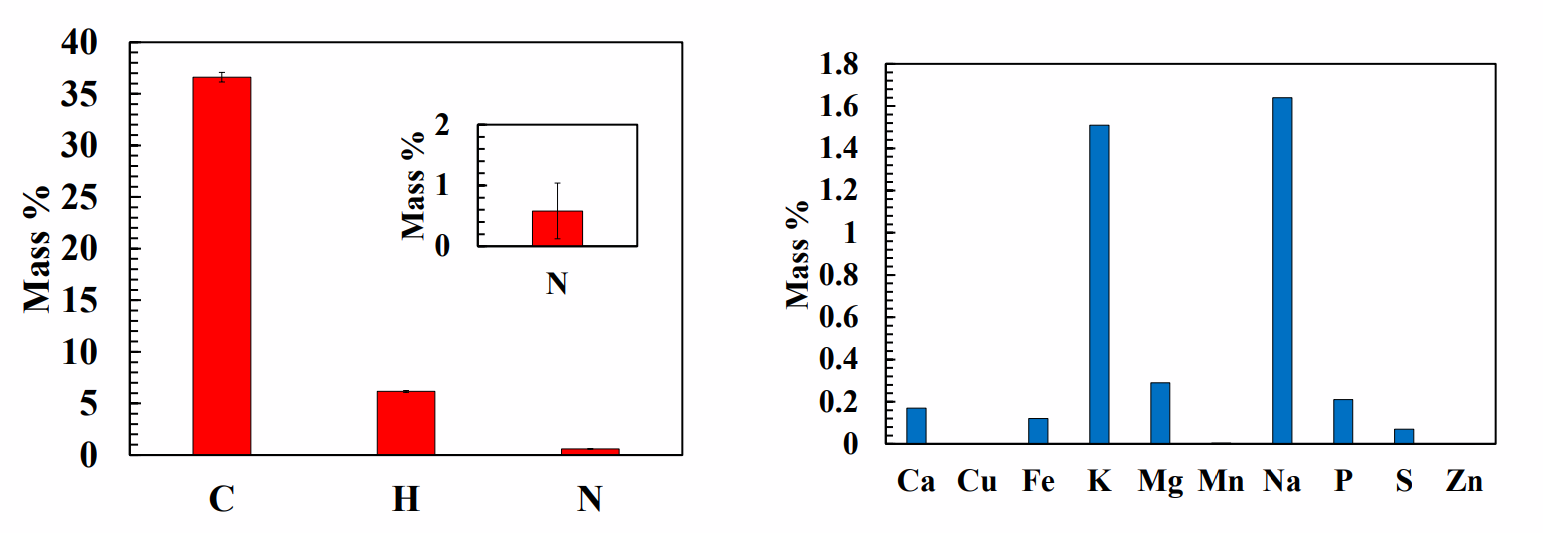


**Figure S1.** Elemental abundances obtained through CHN (Exeter Analytical CE 440) and ICP microanalysis (PerkinElmer NexION 350D) of sorghum hydrolysate prepared using 50 mM citrate buffer. The calculated C/N mass ratio was 63.
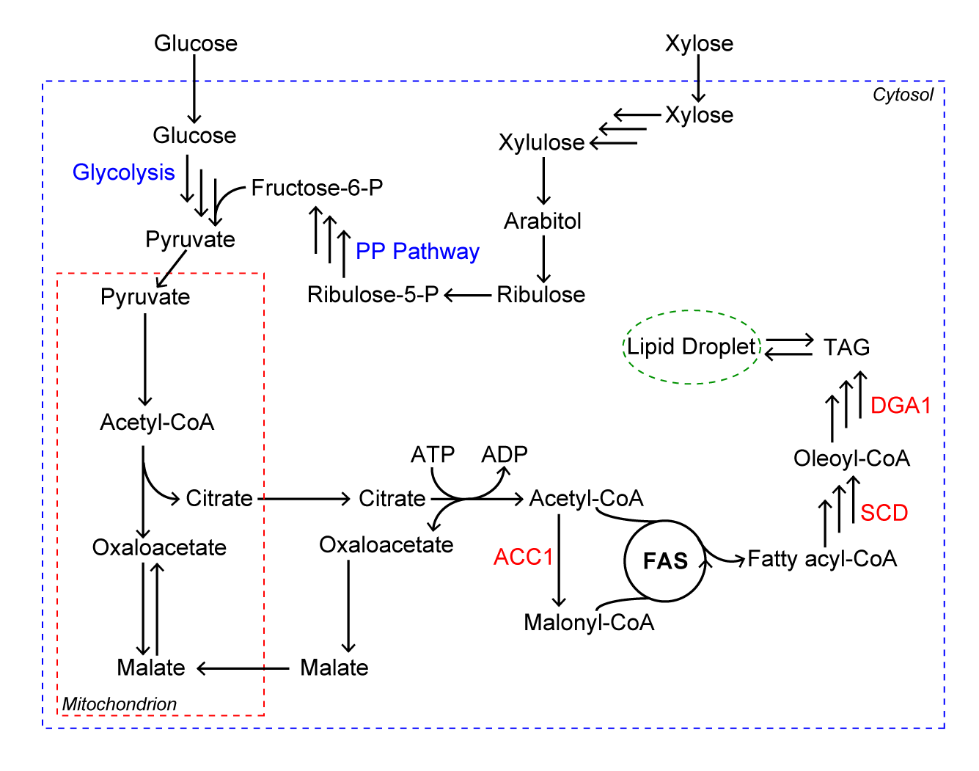


**Figure S2.** Pathway diagram of glucose and xylose utilization and conversion to lipids in *R. toruloides*. Enzymes overexpressed in the RT-ADS strain are labeled in red.

**Table S1.** Strain information

| **Strain** | **Genotype** | | | **Source** |
| --- | --- | --- | --- | --- |
| IFO0880 | | *Rhodosporidium toruloides* strain IFO0880; mating type a | NBRC collection | |
| RT880-ADS | | IFO0880/P_GAPDH_ -ACC1-T_ACC1_-P_ACL_-DGA1-T_DGA1_-pGI2H-SCD | (Zhang et al. 2016) | |

**References**

Zhang S, Ito M, Skerker JM, Arkin AP, Rao CV. Metabolic engineering of the oleaginous yeast *Rhodosporidium toruloides* IFO0880 for lipid overproduction during high-density fermentation. *Appl Microbiol Biotechnol.* 2016;100(21):9393–405.
